# Personalized radiomics signature to screen for KIT-11 mutation genotypes among patients with gastrointestinal stromal tumors: a retrospective multicenter study

**DOI:** 10.1186/s12967-023-04520-w

**Published:** 2023-10-16

**Authors:** Qing-Wei Zhang, Ran-Ying Zhang, Zhi-Bo Yan, Yu-Xuan Zhao, Xin-Yuan Wang, Jing-Zheng Jin, Qi-Xuan Qiu, Jie-Jun Chen, Zhen-Hui Xie, Jiang Lin, Hui Cao, Yan Zhou, Hui-Min Chen, Xiao-Bo Li

**Affiliations:** 1 Division of Gastroenterology and Hepatology, Shanghai Institute of Digestive Disease, NHC Key Laboratory of Digestive Diseases, State Key Laboratory for Oncogenes and Related Genes, Renji Hospital, School of Medicine, Shanghai Jiao Tong University, Shanghai, China; 2 Department of Radiology, Zhongshan Hospital, Fudan University, Shanghai, 200032 China; 3 Department of General Surgery, Qilu Hospital of Shandong University, Jinan, China; 4 Department of Radiology, Qilu Hospital of Shandong University, Jinan, China; 5 Department of Gastrointestinal Surgery, Renji Hospital, Shanghai Jiao Tong University School of Medicine, No. 160 Pujian Road, Shanghai, 200127 China; 6 Department of Radiology, Renji Hospital, School of Medicine, Shanghai Jiao Tong University, No. 160, Pujian Rd., Shanghai, 200127 China

**Keywords:** Radiomics, Computed tomography, Gastrointestinal stromal tumor (GIST), Imaging genomics, Mutation, KIT exon 11

## Abstract

**Objectives:**

Gastrointestinal stromal tumors (GISTs) carrying different KIT exon 11 (KIT-11) mutations exhibit varying prognoses and responses to Imatinib. Herein, we aimed to determine whether computed tomography (CT) radiomics can accurately stratify KIT-11 mutation genotypes to benefit Imatinib therapy and GISTs monitoring.

**Methods:**

Overall, 1143 GISTs from 3 independent centers were separated into a training cohort (TC) or validation cohort (VC). In addition, the KIT-11 mutation genotype was classified into 4 categories: no KIT-11 mutation (K11-NM), point mutations or duplications (K11-PM/D), KIT-11 557/558 deletions (K11-557/558D), and KIT-11 deletion without codons 557/558 involvement (K11-D). Subsequently, radiomic signatures (RS) were generated based on the arterial phase of contrast CT, which were then developed as KIT-11 mutation predictors using 1408 quantitative image features and LASSO regression analysis, with further evaluation of its predictive capability.

**Results:**

The TC AUCs for K11-NM, K11-PM/D, K11-557/558D, and K11-D ranged from 0.848 (95% CI 0.812–0.884), 0.759 (95% CI 0.722–0.797), 0.956 (95% CI 0.938–0.974), and 0.876 (95% CI 0.844–0.908), whereas the VC AUCs ranged from 0.723 (95% CI 0.660–0.786), 0.688 (95% CI 0.643–0.732), 0.870 (95% CI 0.824–0.918), and 0.830 (95% CI 0.780–0.878). Macro-weighted AUCs for the KIT-11 mutant genotype ranged from 0.838 (95% CI 0.820–0.855) in the TC to 0.758 (95% CI 0.758–0.784) in VC. TC had an overall accuracy of 0.694 (95%CI 0.660–0.729) for RS-based predictions of the KIT-11 mutant genotype, whereas VC had an accuracy of 0.637 (95%CI 0.595–0.679).

**Conclusions:**

CT radiomics signature exhibited good predictive performance in estimating the KIT-11 mutation genotype, especially in prediction of K11-557/558D genotype. RS-based classification of K11-NM, K11-557/558D, and K11-D patients may be an indication for choice of Imatinib therapy.

**Supplementary Information:**

The online version contains supplementary material available at 10.1186/s12967-023-04520-w.

## Introduction

Subepithelial cancers of the gastrointestinal system are most common in the form of gastrointestinal stromal tumors (GISTs) [1, 2]. Malignant and noncancerous GISTs may be diagnosed, with surgery being the treatment of choice [1, 2]. In addition, small molecule tyrosine kinase inhibitors (TKIs), particularly Imatinib, are known to significantly boost the prognosis of GISTs [3].

Gain-of-function mutations in c-kit (KIT) or platelet-derived growth factor receptor alpha (PDGFRA) receptor tyrosine kinase genes are frequent in individuals with GISTs [4–7]. The 95% of adult GIST patients have abnormally high levels of KIT protein, and 80% of GIST patients have mutations in the KIT gene [4–7]. Exon 11, which codes for the intracellular juxtamembrane region of the KIT receptor, is the most common site for mutations in the KIT gene [4–7]. Interestingly, Point mutations (PM), deletions (D), and insertions (I) in KIT exon 11 have all been observed, which is rather intriguing (I). Most GISTs have been linked to a gain-of-function mutation in the tyrosine kinase function of c-kit, suggesting a role for this mutation in the etiology of this tumor [8, 9].

Given its strong prevalence and poor prognosis with late detection, it is essential to identify GIST mutation biomarkers which can aid in diagnosis and personalized treatment planning. It was previously revealed that the GIST response to targeted therapy and its disease progression is highly dependent on the location and form of genetic mutation [9–12]. According to one research, GIST with a mutation in the main KIT exon 11 had the best response to Imatinib [13]. However, GIST with mutations in exon 17 of the KIT gene or exon 18 of the PDGFRA gene is resistant to Imatinib [14, 15].

GIST prognosis determination depends on responses to targeted therapy. Prior reports suggested that GISTs carrying varying primary KIT exon 11 mutations give rise to distinct patient prognoses. Although there is no general consensus regarding gene mutation and prognosis association, several clinical trials demonstrated that D, particularly, KIT exon 11 codon 557-558 D (K11-557/558D) is linked to disease progression and the largest postsurgical recurrence rate among GIST patients. However, these patients respond well to Imatinib [9, 11, 16]. Given this evidences, longer postsurgical targeted therapy was recommend for these patients [10, 17].

Typically, surgically removed tissue samples are used for GIST gene mutation analysis. Unfortunately, some patients with GIST are diagnosed with tumor metastases or rather big tumors, making surgical excision impossible. It is possible to use fine-needle aspiration to acquire tissue for pathological assessment. However, the extracted sample is generally insufficient for genotyping. Moreover, routine genotyping is also avoided owing to its relatively high cost, even among surgical resection patients.

Medical imaging is a robust tool with multiple applications, including, disease diagnosis and treatment guidance [18]. It is commonly used owing to its non-invasive nature and relatively thorough assessment of the internal tissues and organs. GIST is frequently identified using computed tomography (CT) [3]. Radiomics allows the conversion of CT scans into high-throughput quantitative data, which may be used to characterize intra-tumor heterogeneity and its possible connections with genetic profiles. The higher effectiveness of radiomics in predicting malignancy and the ki-67 profile among GIST patients has been shown in recent papers [19, 20]. Radiogenomics integrates clinical imaging information with molecular and genomic imaging [21]. Multiple recent investigations reported strong correlations between tumor radiomics and gene profiles belonging to renal cell carcinoma, breast cancer, gliomas, neck tumors and GISTs [18, 22–24]. Few studies also examined the feasibility of employing radiogenomics to study KIT-11 mutation among GIST patients [25, 26]. However, these studies only investigated whether radiomics can estimate KIT-11 mutation among GIST patients, and they did not differentiate between varying KIT-11 mutation genotypes, which, as we mentioned earlier, produces distinct disease progressions, postoperative recurrence rates, and responses to Imatinib [9, 11, 16].

Herein, we separated eligible GIST patients into 4 categories: no mutation (K11-NM), K11-PM/D, K11-557/558D, and KIT-11 D not involving codons 557/558 (K11-D). We explored the predictive performance of our radiomics signature extracted from the arterial phase of contrast-corrected CT to predict varying KIT-11 mutation genotypes.

## Methods

### Patient population

The institutional review board approved the study protocol, and the study was conducted in accordance with ethical principles of the 1975 Declaration of Helsinki and subsequent revisions (KY2023-002-B). Consent requirement have been waved by institutional review board due to its retrospective study. Overall, 1143 GIST patients were enrolled from 3 medical centers for this retrospective investigation. The following patients were selected for analysis: (1) those who received surgery; (2) standard contrast-enhanced CT (CE-CT) < 2 weeks prior to treatment; (3) histology- and immunohistochemistry-based GIST diagnosis; (4) available previously analyzed clinical and pathological variables. Among those that were eliminated from analysis were patients with prior Imatinib treatment or numerous GISTs or cases involving inadequate image quality (e.g., missing contrast-enhanced CT portal phase, severe motion artifact).

**Fig. 1:**
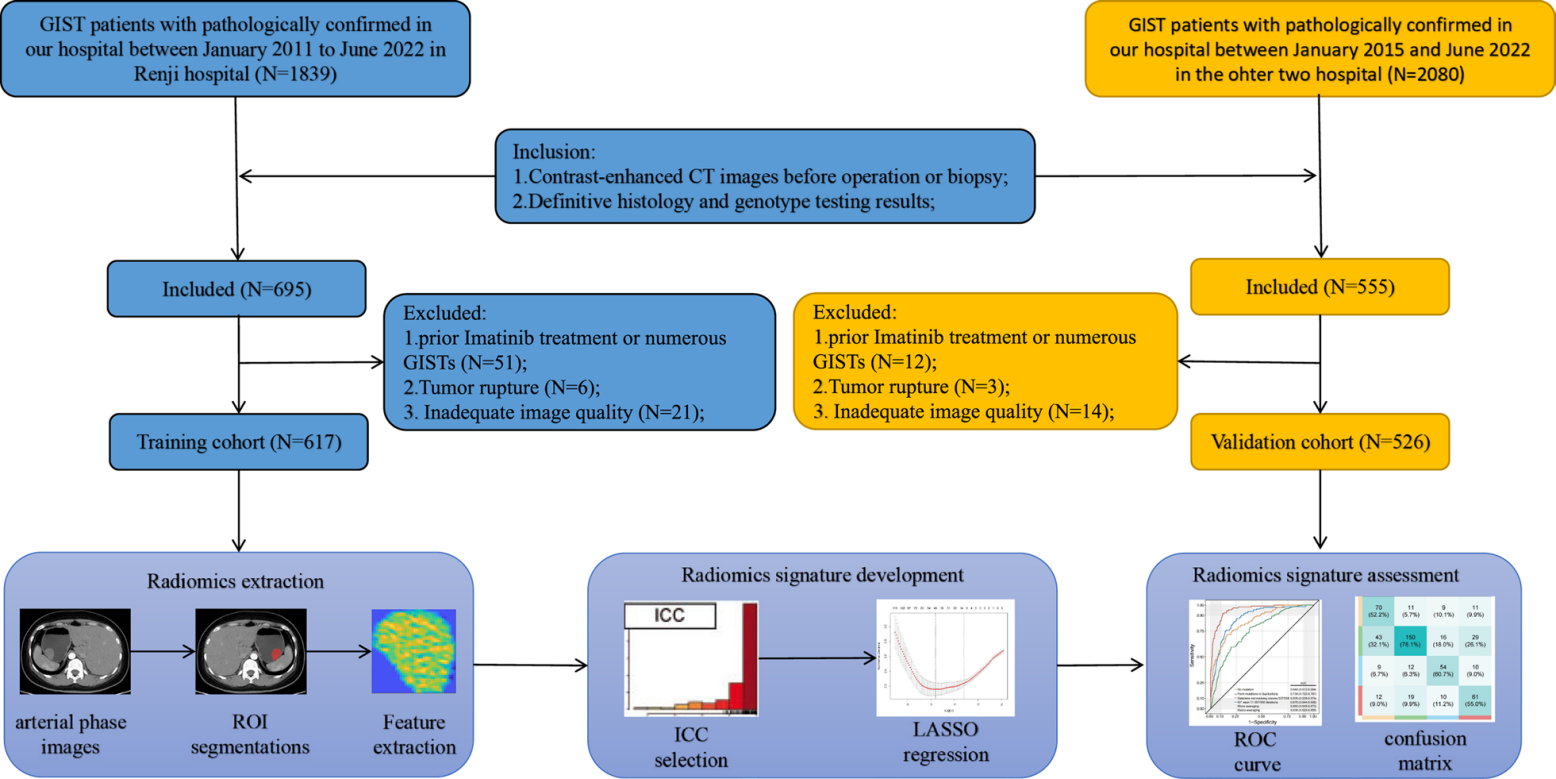
Research design. GIST, gastrointestinal stromal tumor; CT, computed tomography; ROI, region of interest; LASSO, least absolute shrinkage and selection operator; ICC, intra- and inter-class correlation coefficients; ROC, receiver operating characteristic

The study subjects were separated into two distinct cohorts, namely, training (TC) and validation cohorts (VC). Between January 2011 and June 2022, 617 patients were chosen from the one hospital for the TC. We chose GIST patients from the remaining two facilities between January 2015 and June 2022 for the VC. The detail of inclusion of GIST patients and radiomcis extraction was shown in the Fig. 1.

### CT assessment

The CT protocol is presented in detail in Additional file 1: A1 and Table S1, which have been validated in the assessment of prediction of Ki-67 expression and maligiant potential in GISTs [19, 27].

### Clinical variable and primary endpoint

We assessed clinical and pathological information, namely, age, gender, tumor site, mitotic count, tumor size, and KIT-11 mutation genotype. The maximum diameter on axial CT scans was used to determine the tumor size. There were four distinct categories for the KIT-11 mutant genotype: K11-NM, K11-PM/D, K11-557/558D, and K11-D. Our primary endpoint was the accuracy in KIT-11 mutation genotype prediction.

### Radiomic signature (RS) construction

ITK-SNAP (version 2.2.0; www.itksnap.org) was used to manually pick the area of interest from all contrast-corrected CT images for each GIST that were downloaded from the Picture Archiving and Communication System. Each patient's CE-CT arterial phase slice pictures were reviewed, and the slice with the most tumor was selected for further examination. After that, for each individual being studied, a 2D region of interest (ROI) with largest area was selected. Our research methodology is shown in Fig. 1.

Each GIST's radiomic profile was obtained using the aforementioned ROI using PyRadiomics in Python (version 3.7), and the resulting profile included first-order statistics, 2D shape features, a grey-level co-occurrence matrix (GLCM), a grey-level run-length matrix (GLRLM), a grey-level size-zone matrix (GLSZM), a gray-level dependence matrix (GLDM), and a neighboring gray-tone difference matrix (NGTDM) [28]. In-depth summaries of these radiomic profiles are provided in Table S2 of the Additional file 1. After that, we followed these steps (Additional file 1: A3) to pick a radiomic profile and build an RS: The ICCs (intra- and inter-class correlation coefficients) [29] are used to assess the repeatability of a profile, whereas the LASSO technique is used to build a RS [30]. In GIST patients, the RS produced the genotype of the KIT-11 mutation. The LASSO coefficients were used to assign relative importance to each radiomics profile, and then RS was calculated as: Rad-score = a_1_X_1_ + a_2_X_2_ + ⋯ + a_n_X_n_ + b.

### Statistical analysis

Categorical variables are stated as raw numbers or percentages, whereas continuous variables are shown as means ± standard deviations or medians and ranges. The t- or Mann–Whitney test for continuous data, and the chi-square test or Fisher's exact test for categorical data, were used to find statistically significant differences in TC and VC across several groups.

Cohen's k statistic was used for both inter- and intra-observer ICCs to assess the level of agreement amongst CT profile readers. Values of kappa over 0.80 indicated very strong agreement, while values between 0.40 and 0.80 indicated moderate correlation, and values below 0.40 indicated minimal consensus.

Receiver operating characteristic (ROC) curves and area under the ROC curve (AUC) with 95% confidence interval (CI) were used to evaluate the precision of the RS predictions, as has been previously reported [31, 32]. In addition, we calculated and showed the TC and VC's prediction accuracy, sensitivity, specificity, negative predictive value (NPV), and positive predictive value (PPV). Micro- and macro-averages are also provided. The micro-average value represents the average instance-level performance. As such, it can form a bias toward the label with the largest frequency count. This is likely why the associated values are relatively high. The macro-averaged value represents the mean performance across all labels. Hence, it provides an enhanced understanding of a model’s performance across different labels [33]. Moreover, the bootstrap technique (N = 1000) was employed for the macro- and micro-averaged value calculations.

R (3.5.0) and Python (3.7) were used for all statistical analyses. The cutoff point for significance was determined to be a P value of 0.05 or below.

## Results

### Clinical baseline profiles of the TC and VC

Overall, 1143 GISTs patients with definitive KIT-11 mutation genotype testing results from 3 centers were separated into TC and VC. TC consisted of 617 GIST patients from one hospital, and VC consisted of 526 GIST patients from the remaining two hospitals. Patients in both groups showed similar demographics (Table 1), including gender, age, geographic region, aggressive behavior risk, and KIT-11 mutant genotype. However, a higher percent of high mitotic count (> 10/50 HPF) was observed in the VC, compared to the TC.

**Table 1:** Clinical characteristics of patients in the training and validation cohort

	Total (n = 1143)	Training (n = 617)	Validation (n = 526)	p
*Sex, n (%)*				0.2
Female	536 (46.89)	296 (47.97)	240 (45.63)	
Male	607 (53.11)	321 (52.03)	286 (54.37)	
Age, Mean ± SD	61 ± 11.7	61.6 ± 12.2	60.4 ± 11.1	0.075
*Location, n (%)*				0.014
Stomach	756 (66.14)	388 (62.88)	368 (69.96)	
Non-stomach	387 (33.86)	229 (37.12)	158 (30.04)	
*Mitotic count (/50 HPF), n (%)*				0.004
< 5	897 (78.48)	503 (81.52)	394 (74.9)	
6 ~ 10	146 (12.77)	75 (12.16)	71 (13.5)	
> 10	100 (8.75)	39 (6.32)	61 (11.6)	
Risk of aggressive behavior*, n (%)				0.322
Very low	77 (6.74)	49 (7.94)	28 (5.32)	
Low	481 (42.08)	256 (41.49)	225 (42.78)	
Intermediate	247 (21.61)	128 (20.75)	119 (22.62)	
High	338 (29.57)	184 (29.82)	154 (29.28)	
*KIT-11 mutation*				0.494
No mutation	268 (23.45)	134 (21.72)	134 (25.48)	
Point mutations or duplications	435 (38.06)	243 (39.38)	192 (36.5)	
Deletions not involving codons 557/558	197 (17.24)	108 (17.5)	89 (16.92)	
KIT exon 11 557/558 deletions	243 (21.26)	132 (21.39)	111 (21.1)	

HPF, high-power field

*According to the modified 2008 National Institute of Health criterion

### Development of RS in prediction of KIT-11 mutation subtyping in TC

Overall, 726 radiomics characteristics with ICC values > 0.8 in the intra- and inter-individual comparisons were employed for model construction. Using LASSO regression, 46, 55, 50, and 39 radiomics were employed for RS construction to predict K11-NM, K11-PM/D, K11-557/558D, and K11-D, respectively. The detailed LASSO coefficients for each radiomics are described in Table S2.

**Fig. 2:**
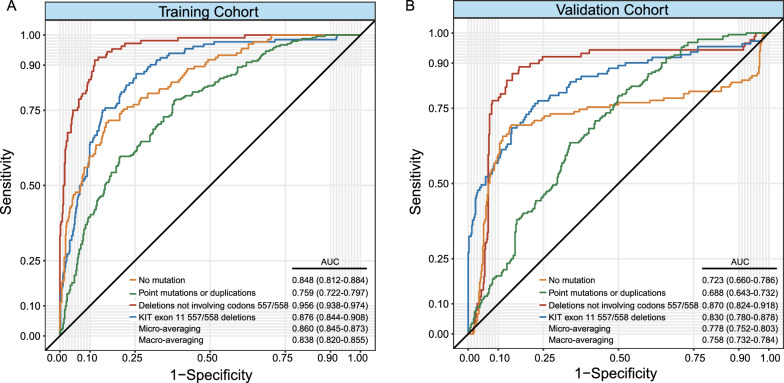
The area under curves (AUCs) of radiomics signature (RS) for prediction of no mutation (K11-NM), point mutation or deletion (K11-PM/D), KIT-11 557/558 deletions (K11-557/558D), KIT-11 deletion not involving codons 557/558 (K11-D), macro-averaging, and micro-averaging in the training (TC) (**A**) and validation cohorts (VC) (**B**)

**Fig. 3:**
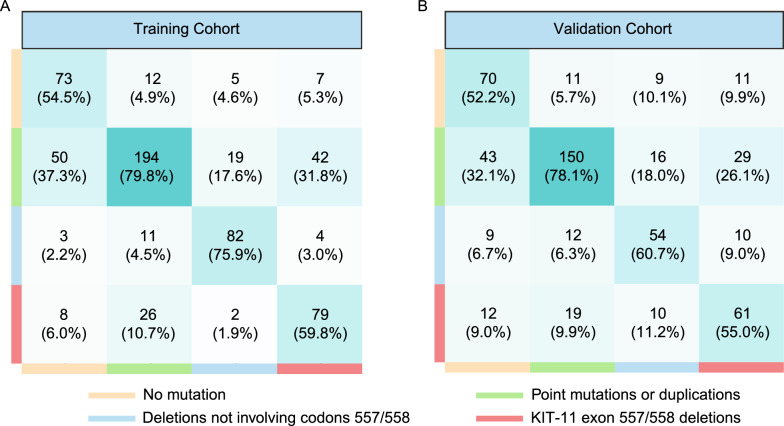
The confusion matrix of diagnosing four-level classification of KIT-11 mutation, involving no mutation (K11-NM), point mutation or deletion (K11-PM/D), KIT-11 557/558 deletions (K11-557/558D), and KIT-11 deletion not involving codons 557/558 (K11-D) in the training (TC) (**A**) and validation cohorts (VC) (**B**)

Our newly constructed RS showed a high AUC in predicting each KIT-11 mutation, as shown in Fig. 2A. The AUCs for K11-NM, K11-PM/D, K11-557/558D, and K11-D predictions were 0.848 (95% CI 0.812–0.884), 0.759 (95% CI 0.722–0.797), 0.956 (95% CI 0.938–0.974), and 0.876 (95% CI 0.844–0.908) respectively. Figure 3A depicts the TC RS confusion matrix. We found that the RS correctly predicted 73 of 114 instances of K11-NM, 194 of 243 cases of K11-PM/D, 79 of 132 cases of K11-557/558D, and 82 of 108 cases of K11-D. We also calculated the diagnostic accuracy of RS for identifying different types of KIT-11 mutations. Table 2 shows the RS specificity ranged from 0.706 (95% CI 0.660–0.752) to 0.965 (95% CI 0.949–0.981), sensitivity ranged from 0.545 (95% CI 0.460–0.629) to 0.798 (95% CI 0.748–0.849), accuracy ranged from 0.742 (95% CI 0.708–0.777) to 0.930 (95% CI 0.910–0.950), NPV ranged from 0.843 (95% CI 0.803–0.884) to 0.965 (95% CI 0.949–0.981) and PPV ranged from 0.638 (95% CI 0.584–0.692) to 0.822 (95% CI 0.747–0.896).

**Table 2:** Diagnostic efficacy of radiomics signature in prediction of KIT-11 mutation genotype in the training and validation cohort

	Training cohort						Validation cohort					
	No mutation	Point mutations or duplications	deletions not involving codons 557/558	KIT exon 11 557/558 deletions	Micro averaging	Macro averaging	No mutation	Point mutations or duplications	deletions not involving codons 557/558	KIT exon 11 557/558 deletions	Micro averaging	Macro averaging
Accuracy	0.862 (0.835–0.889)	0.741 (0.706–0.775)	0.929 (0.908–0.949)	0.856 (0.828–0.883)	0.781 (0.755–0.808)	0.694 (0.66–0.729)	0.819 (0.787–0.852)	0.753 (0.716–0.79)	0.875 (0.846–0.903)	0.827 (0.795–0.859)	0.743 (0.714–0.772)	0.637 (0.595–0.679)
Sensitivity	0.545 (0.46–0.629)	0.798 (0.748–0.849)	0.759 (0.679–0.84)	0.598 (0.515–0.682)	0.694 (0.656–0.734)	0.694 (0.66–0.729)	0.522 (0.438–0.607)	0.781 (0.723–0.84)	0.607 (0.505–0.708)	0.55 (0.457–0.642)	0.637 (0.593–0.681)	0.637 (0.595–0.679)
Specificity	0.95 (0.931–0.97)	0.703 (0.657–0.75)	0.965 (0.949–0.981)	0.926 (0.902–0.949)	0.898 (0.885–0.911)	0.85 (0.828–0.869)	0.921 (0.894–0.948)	0.737 (0.689–0.784)	0.929 (0.905–0.953)	0.901 (0.872–0.93)	0.879 (0.864–0.894)	0.851 (0.831–0.871)
PPV	0.753 (0.667–0.838)	0.636 (0.582–0.69)	0.82 (0.745–0.895)	0.687 (0.602–0.772)	0.694 (0.656–0.734)	0.704 (0.671–0.74)	0.693 (0.603–0.783)	0.63 (0.569–0.692)	0.635 (0.533–0.738)	0.598 (0.503–0.693)	0.637 (0.593–0.681)	0.639 (0.6–0.683)
NPV	0.883 (0.855–0.91)	0.843 (0.803–0.883)	0.95 (0.931–0.969)	0.894 (0.868–0.921)	0.898 (0.885–0.911)	0.881 (0.861–0.897)	0.849 (0.815–0.883)	0.854 (0.813–0.895)	0.921 (0.895–0.946)	0.882 (0.851–0.913)	0.879 (0.864–0.894)	0.87 (0.851–0.887)

### Validation of RS in prediction of KIT-11 mutation subtyping in VC

We next validated our newly developed RS in the VC from two medical centers. The AUCs were 0.723 (95% CI 0.660–0.786), 0.688 (95% CI 0.643–0.732), 0.870 (95% CI 0.824–0.918), and 0.830 (95% CI 0.780–0.878) for K11-NM, K11-PM/D, K11-557/558D, and K11-D prediction, respectively. The RS confusion matrix for VC is depicted in Fig. 3B. Based on our observation, there were 70 (52.2%) cases of K11-NM, 150 (78.1%) cases of K11-PM/D, 61 (55.0%) cases of K11-557/558D, and 54 (60.7%) cases of K11-D which were accurately predicted by the RS in VC. We also computed the RS diagnostic efficacy in predicting each KIT-11 mutation class in the VC. As depicted in Table 2, the RS specificity ranged from 0.737 (95% CI 0.689–0.784) to 0.929 (95% CI 0.905–0.953), sensitivity ranged from 0.522 (95% CI 0.438–0.607) to 0.781 (95% CI 0.723–0.840), NPV ranged from 0.854 (95% CI 0.813–0.895) to 0.921 (95% CI 0.895–0.946), PPV ranged from 0.598 (95% CI 0.503–0.693) to 0.693 (95% CI 0.603–0.783), and accuracy ranged from 0.637 (95% CI 0.595–0.610) to 0.875 (95% CI 0.846–0.903).

As depicted in Fig. 2, the micro-averaging AUCs were 0.860 (95% CI 0.845–0.873) and 0.778 (95% CI 0.752–0.803) in the TC and VC. The macro-averaging AUCs were 0.838 (95% CI 0.820–0.855) and 0.758 (95% CI 0.758–0.784) in the TC and VC. In terms of the macro- and micro-averaging diagnostic efficacies, the micro-averaging accuracies were 0.781 (95% CI 0.755–0.808), and 0.694 (95% CI 0.660–0.729) in the TC and VC, respectively. The macro-averaging accuracies were 0.694 (95% CI 0.660–0.729), and 0.637 (95% CI 0.595–0.679) in the TC and VC, respectively. The micro- and macro-averaging values are detailed in Table 2.

## Discussion

Herein, we explored the feasibility of the radiomics profile to predict varying KIT exon 11 K11-Mutations in GISTs using various contrast-corrected CT images from large-scale imaging data. We established a four-level classification model with satisfactory performance to probe the KIT-11 mutation genotype profiles of GISTs, based on contrast CT images. We further demonstrated that our newly developed RS can accurately predict the KIT-11 mutation genotype.

Historically, the application of medical imaging was primarily driven by necessity. The advent of radiomics has revolutionized this approach, enabling the conversion of medical images into high-throughput quantitative data that may be linked to factors such as intra-tumor heterogeneity and individual patient genetics (radiogenomics). In 2018, Xu et al. conducted a study that showcased the potential of CT texture analysis of enhanced CT images to differentiate between GIST without K11-mutation and GIST with K11-mutation [25]. They further proposed that the standard deviation of tumor texture parameters could serve as a unique indicator of GIST without K11-D. While this study was a significant contribution to the field, it should be noted that it was conducted with a relatively small patient population, consisting of 69 cases in TC and 17 cases in VC. Additionally, the VC group only included 4 cases of GIST without K11-mutation, which could potentially have influenced the results. The study also limited its retrieval to 30 radiomics characteristics from CT images for texture analysis [25]. These limitations were addressed in a subsequent study by Liu et al. [26]. However, it's important to note that this was a single-center study, and its primary focus was to predict whether the GIST involved K11-mutation or not. It's crucial to appreciate the contributions of each study while also acknowledging their limitations. These limitations do not detract from the value of the research but rather provide avenues for further exploration and improvement in future studies.

The current investigation does not have the deficiencies described in the above two studies, and it shows significant progress. First, we employed a significantly larger patient population. We trained RS using 617 GIST patients, and externally validated the RS in two independent medical centers. Second, we analyzed 1408 radiomics features for radiogenomics, which is considerably more than the above two studies. Third, unlike the aforementioned studies, we predicted a four-level KIT-11 mutation genotype classification using RS, and produced satisfactory results.

We demonstrated that our newly developed RS may be economical for usage in clinics to guide Imatinib treatment planning and outcome monitoring. Of note, in terms of an RS-based classification of GIST with K11-PM/D, clinicians must be cautious about starting standard Imatinib therapy, as only 63.6% of estimated K11-PM/D in the TC and 63.0% in the VC was histologically confirmed to be true KIT-11 K11-PM/D. Thus, for these patients, selective genotype testing may be a better choice to guide targeted treatment rather than unselective standard Imatinib therapy. Moreover, it is suggested that patients initially classified as GISTs without K11-mutation using RS are also sent for genotype testing for the identification of other potential mutations, for example, KIT-9, 13, 17 mutation or PDGFRA-12, 18 mutation [9–12]. Alternately, the RS-based classification of K11-NM, K11-557/558D, and K11-D patients can be treated with Imatinib therapy and prognosis monitoring can occur according to the predicted KIT-11 mutation type using RS.

This study encountered certain limitations. First, being a retrospective research and with our strict exclusion criteria, the study may have introduced unintentional selection bias. Second, owing to the relatively small patient population in certain KIT or PDGFRA mutation, we were unable to explore the RS feasibility in predicting KIT-9, KIT-12 and PDGFRA mutation. Thus, we only grouped GISTs KIT-11 mutation into four-level classification. However, we are aware that it is imperative to distinguish between some of these mutations, for example, small intestinal GIST with K11-mutation from the KIT exon 9 (K9) mutation, which may be associated with poorer response to targeted therapy and worse prognosis. This must be addressed in future well-designed investigations with large population cohort. Third, we assessed and demonstrated a correlation between GIST with K11-D and contrast-corrected CT imaging. However, the underlying biochemical and clinical mechanisms of this correlation were not explored in this study. In addition, the mitotic count of GISTs was different in the TC and the VC, and we used scanners from three separate locations to determine this. Our aim is that this would lead to widespread use of our models since they are replicable and reliable. Forth, though segmentation was done by an experienced radiologist in 2D to ensure accuracy of segmentation, but this process could be really time-consuming which may again limit the clinical implementation. In the future, automatic segmentation and automatic calculation of probality of different KIT-11 mutation could be tested. Finally, the ROIs were chosen in a single slice (2D), which may not provide an accurate depiction of the complete tumor. Moreover, certain radiomics characteristics, for instance, the texture profile, may be impacted when retrieved from 2D, and not 3D, imaging. Hence, it is critical to perform the 3D analyses of the entire GIST in the future.

## Conclusion

It is indicated that contrast-corrected CT imaging may be useful for prediction of KIT-11 mutation genotype given further evaluation, especially in prediction of K11-557/558D genotype. Our automated feature algorithms could facilitate further investigation using the image-based quantitative features. Given that CT imaging is widely employed all over the world, tapping into its rich data for GIST stage diagnoses and treatment can be extremely beneficial for clinicians and patients, and it can potentially enhance Imatinib therapy and GIST monitoring. RS-based classification of K11-NM, K11-557/558D, and K11-D patients may be an indication for choice of Imatinib therapy.

### Supplementary Information

**Additional file 1**: CT assessment and Radiomic signature development.

## Data Availability

The data generated in this study are available upon request from the corresponding author.
