# Supplementary material for: Personalized radiomics signature to screen for KIT-11 mutation genotypes among patients with gastrointestinal stromal tumors: a retrospective multicenter study
Source: J Transl Med. 2023 Oct 16;21:726. doi: 10.1186/s12967-023-04520-w (PMC10577986; doi:10.1186/s12967-023-04520-w)
Supplement: Supplementary file 1 — Additional file 1: CT assessment and Radiomic signature development. [file 12967_2023_4520_MOESM1_ESM.docx]

**Supplementary A1: CT examinations**

Enrolled patients in the three centers underwent similar scan setup but with different systems and parameters. For stomach scan, all patients were recommended to be overnight fasted and 20 mg of scopolamine or anisodamine were administered intramuscularly to reduce gastrointestinal peristalsis 15–20 min prior to computed tomography (CT) examination. For small bowel scan, all patients were recommended to be overnight fasted with 2L or 3L polyethylene glycol (PEG) before the day of examination or 1L to 2L mannitol on the day of examination. For large bowel scan, all patients were recommended to be fasted for 4 to 6 hours on the day of examination. Patients were asked to drink 600–1000 ml warm water or received 6 g of effervescent granules to distend the stomach prior to CT examination.

The CT scans, covering the entire stomach region or bowel region, were acquired during a breath-hold with the patient supine. The CT image acquisition parameters of the three centers were shown in Table S1. The diagnosis was performed with a standardized dynamic window adjustment procedure, on window-adjustable PACS work stations. A narrow window was used to demonstrate the primary GIST. Three-plane images (axial, coronal, and sagittal) were observed together to facilitate the detection and location of the primary GIST.

**Supplementary A2: Radiomic features extraction.**

Before extraction of radiomic features, all arterial phase CT images were subjected to imaging normalization (the intensity of the image was scaled to 0–500) and resampled to the same resolution (1mm×1mm×1mm) before feature extraction to avoid data heterogeneity bias.

After that, filtering process was performed to implement image smoothing and image difference before CT radiomic feature extraction, including Wavelet, Square, SquareRoot, Logarithm, Exponential, Gradient, LocalBinaryPattern2D filters. Separable filtering was used to avoid the multi-dimensional convolution. The wavelet convolution was performed with a low-/high-pass “Coiflet 1” wavelet filter along the x-/y-direction by applying different weights to band-pass and sub-bands (LHL, LHH, LLH, HLL, HHL and HLH) of the tumor region as compared to low frequency and high frequency sub-bands (LLL and HHH) in the wavelet domain.

After filtering, a total of 1408 quantitative features were extracted from the ROI of the original image and its corresponding filtered results, including 18 features of first order statistics, 14 features of shape, 24 features of grey-level co-occurrence matrix (GLCM), 16 features of grey-level run-length matrix (GLRLM), 16 features of grey-level size- zone matrix (GLSZM), 14 gray level dependence matrix GLDM), 5 features of neighbouring gray tone difference matrix (NGTDM) and 1301 features from images with filtering. The process of image filtering and feature extraction was performed using PyRadiomics on Python (3.7) (1).

Radiomic features of all patients were standardized by the z-score method, based on the parameters calculated from the training cohort.

**Supplementary A3: Radiomic feature selection and signature building process**

One radiologist with 9 years of experience with CT (reader 1) performed segmentations for all patients. Three months after the initial segmentation, 40 patients in the training cohort were randomly selected and re-segmented by this radiologist to assess intra-reader agreement in radiomic features. These cases were then segmented by another radiologist with 5 years of experience with CT (reader 2) for inter-reader agreement assessment.

The feature selection and signature building process were performed for three steps. First, based on the different independent segmentation groups, intra-/inter-class correlation coefficients (ICCs) were used to estimate each feature’s reproducibility (2). Stable features with ICCs >0.8 were reserved. For radiomic signature building, the Least Absolute Shrinkage and Selection Operator Method (LASSO) logistic regression model was used to build Radiomics signature (RS) (3).

**Table S1.** The CT protocol of the three centers

| Parameters | Center 1 | Center 2 | Center 3 |
| --- | --- | --- | --- |
| CT version | Spectral CT (Discovery CT750 HD scanner, GE Healthcare, USA) | Spectral CT (Aquilion One TSX-301A, TOSHIBA, Japan) | Philips Brilliance 256-slice helical CT scanner (Brilliance ICT, Philips, Netherlands) |
| CT  tube voltage | 120 kVp | 120 kVp | 120 kVp |
| CT  tube current | 220 mA | 60 mA | 160–600 mA |
| CT  rotation time | 0.6 s | 0.6 s | 0.5 s |
| CT detector collimation | 64×0.625 mm | 64×0.5 mm | 128×0.625 mm |
| Contrast agent type | Iopamidol, Iopamiro, Bracco Sine, Shanghai, China | Iopamiro, Bracco Sine, Shanghai, China | Iopromide, Bayer Medical, Berlin, Germany |
| Contrast agent concentration | 370 mgI/ml | 300 mgI/ml | 350 mg I/mL |
| Contrast agent dosage | infused 1.5 ml/kg body weight | infused 1.5 ml/kg body weight | infused 1.5 ml/kg body weight |
| Contrast agent infused rate | 3.0 ml/s | 3.0 ml/s | 2.5–3.0 mL/s |
| Arterial phase  interval time | 35 s after injection of  contrast agent | 36 s after injection of  contrast agent | 30 s after injection of  contrast agent |
| Venous phase  interval time | 70 s after injection of  contrast agent | 90 s after injection of  contrast agent | 70 s after injection of  contrast agent |
| Image matrix | 512×512 | 512×512 | 512×512 |
| Field of view | 500×500 mm | 500×500 mm | 500×500 mm |
| Reconstruction image thickness | 1 mm | 1 mm | 1 mm |

**Table S2**. Detailed LASSO coefficients for each radiomics for development of the four-level classification of KIT-11 mutation.

| Radiomics | beta | KIT-11 mutaion type |
| --- | --- | --- |
| b | 0.136869704 | No mutation |
| original_shape_Elongation | -0.017320949 | No mutation |
| wavelet.LLH_glcm_Contrast | 0.094411032 | No mutation |
| wavelet.LLH_glcm_Correlation | -0.095302436 | No mutation |
| wavelet.LLH_glcm_DifferenceAverage | 0.001739769 | No mutation |
| wavelet.LLH_glcm_Idmn | -4.73E-08 | No mutation |
| wavelet.LLH_glcm_Idn | -1.85E-08 | No mutation |
| wavelet.LLH_glrlm_HighGrayLevelRunEmphasis | 0.044305438 | No mutation |
| wavelet.LLH_glrlm_LowGrayLevelRunEmphasis | -2.97E-07 | No mutation |
| wavelet.LLH_glrlm_ShortRunLowGrayLevelEmphasis | 0.07957264 | No mutation |
| wavelet.LLH_glszm_ZonePercentage | -0.088935213 | No mutation |
| wavelet.LHL_glszm_GrayLevelNonUniformity | 0.064508174 | No mutation |
| wavelet.LHL_gldm_DependenceEntropy | 0.000155652 | No mutation |
| wavelet.LHL_ngtdm_Coarseness | -1.36E-05 | No mutation |
| wavelet.LHL_ngtdm_Strength | -0.066703879 | No mutation |
| wavelet.LHH_firstorder_90Percentile | -0.133718304 | No mutation |
| wavelet.LHH_firstorder_Kurtosis | 0.009460393 | No mutation |
| wavelet.LHH_glcm_ClusterShade | -0.074887257 | No mutation |
| wavelet.LHH_glszm_ZoneEntropy | -0.090342132 | No mutation |
| wavelet.HLL_glcm_MaximumProbability | 0.046148659 | No mutation |
| wavelet.HLL_glrlm_ShortRunHighGrayLevelEmphasis | 0.178295034 | No mutation |
| wavelet.HLL_glszm_HighGrayLevelZoneEmphasis | -0.030413089 | No mutation |
| wavelet.HLL_gldm_LargeDependenceHighGrayLevelEmphasis | -0.205964498 | No mutation |
| wavelet.HLH_firstorder_Kurtosis | 0.012952269 | No mutation |
| wavelet.HLH_firstorder_Minimum | -0.025895929 | No mutation |
| wavelet.HLH_glrlm_LongRunHighGrayLevelEmphasis | -0.013773357 | No mutation |
| wavelet.HLH_glrlm_RunVariance | -0.05091219 | No mutation |
| wavelet.HLH_glszm_ZoneEntropy | 0.028500009 | No mutation |
| wavelet.HHL_firstorder_Kurtosis | 0.025121124 | No mutation |
| wavelet.HHL_glrlm_ShortRunHighGrayLevelEmphasis | -0.066566543 | No mutation |
| wavelet.HHL_gldm_DependenceNonUniformityNormalized | 0.031813936 | No mutation |
| wavelet.HHL_gldm_HighGrayLevelEmphasis | -0.157054872 | No mutation |
| wavelet.HHL_gldm_LowGrayLevelEmphasis | 6.28E-07 | No mutation |
| wavelet.HHH_glcm_Imc2 | 0.316176179 | No mutation |
| wavelet.HHH_glrlm_RunLengthNonUniformityNormalized | -0.149161952 | No mutation |
| wavelet.HHH_glrlm_ShortRunHighGrayLevelEmphasis | -0.028378889 | No mutation |
| square_firstorder_10Percentile | 0.01418591 | No mutation |
| squareroot_firstorder_90Percentile | -0.024058252 | No mutation |
| squareroot_firstorder_Median | -0.014317903 | No mutation |
| logarithm_firstorder_90Percentile | -0.007351044 | No mutation |
| logarithm_firstorder_Median | -0.052342832 | No mutation |
| lbp.2D_firstorder_10Percentile | -0.42292339 | No mutation |
| lbp.2D_firstorder_Kurtosis | -1.167505111 | No mutation |
| lbp.2D_firstorder_Mean | 0.442648537 | No mutation |
| wavelet.HLL_gldm_SmallDependenceEmphasis | 0.038218377 | No mutation |
| lbp.2D_firstorder_InterquartileRange | -0.943548111 | No mutation |
| lbp.2D_firstorder_RobustMeanAbsoluteDeviation | -1.766364962 | No mutation |
| b | 0.880793039 | Point mutations or duplications |
| original_shape_Elongation | 0.017320949 | Point mutations or duplications |
| original_glszm_LargeAreaHighGrayLevelEmphasis | 0.118738564 | Point mutations or duplications |
| original_ngtdm_Strength | 0.040022279 | Point mutations or duplications |
| wavelet.LLH_firstorder_Skewness | 0.068886012 | Point mutations or duplications |
| wavelet.LLH_glcm_ClusterShade | -0.070208447 | Point mutations or duplications |
| wavelet.LLH_glrlm_ShortRunEmphasis | -0.044779647 | Point mutations or duplications |
| wavelet.LLH_glrlm_ShortRunHighGrayLevelEmphasis | -0.00586803 | Point mutations or duplications |
| wavelet.LLH_glszm_GrayLevelNonUniformity | 0.106709692 | Point mutations or duplications |
| wavelet.LLH_gldm_SmallDependenceLowGrayLevelEmphasis | 0.092650171 | Point mutations or duplications |
| wavelet.LLH_ngtdm_Strength | 0.047354415 | Point mutations or duplications |
| wavelet.LHL_firstorder_Median | 0.150035775 | Point mutations or duplications |
| wavelet.LHL_gldm_SmallDependenceHighGrayLevelEmphasis | -0.002856153 | Point mutations or duplications |
| wavelet.LHH_glcm_DifferenceEntropy | 0.170626978 | Point mutations or duplications |
| wavelet.LHH_glcm_Imc1 | 0.052421902 | Point mutations or duplications |
| wavelet.LHH_glcm_JointAverage | -0.087848812 | Point mutations or duplications |
| wavelet.LHH_glcm_SumAverage | -1.33E-06 | Point mutations or duplications |
| wavelet.LHH_glrlm_ShortRunHighGrayLevelEmphasis | 0.010771108 | Point mutations or duplications |
| wavelet.LHH_glszm_SizeZoneNonUniformity | -0.043392806 | Point mutations or duplications |
| wavelet.LHH_gldm_SmallDependenceLowGrayLevelEmphasis | -0.182096449 | Point mutations or duplications |
| wavelet.HLL_firstorder_Median | 0.021132024 | Point mutations or duplications |
| wavelet.HLL_glcm_ClusterShade | -0.000527798 | Point mutations or duplications |
| wavelet.HLL_glcm_Imc1 | -0.000339381 | Point mutations or duplications |
| wavelet.HLL_glcm_MCC | 0.019115101 | Point mutations or duplications |
| wavelet.HLL_glcm_MaximumProbability | -0.143667339 | Point mutations or duplications |
| wavelet.HLL_glrlm_RunVariance | 0.148570069 | Point mutations or duplications |
| wavelet.HLL_glszm_SmallAreaLowGrayLevelEmphasis | -0.01230491 | Point mutations or duplications |
| wavelet.HLL_gldm_SmallDependenceHighGrayLevelEmphasis | 0.429779527 | Point mutations or duplications |
| wavelet.HLH_firstorder_Kurtosis | 0.0595457 | Point mutations or duplications |
| wavelet.HLH_firstorder_Median | 0.142540633 | Point mutations or duplications |
| wavelet.HLH_firstorder_Minimum | 0.01867936 | Point mutations or duplications |
| wavelet.HLH_firstorder_Skewness | -0.057733898 | Point mutations or duplications |
| wavelet.HLH_glcm_Imc2 | 0.135606712 | Point mutations or duplications |
| wavelet.HLH_glrlm_ShortRunLowGrayLevelEmphasis | 0.102344357 | Point mutations or duplications |
| wavelet.HLH_gldm_SmallDependenceEmphasis | 0.085777184 | Point mutations or duplications |
| wavelet.HHL_glcm_DifferenceEntropy | 0.000912596 | Point mutations or duplications |
| wavelet.HHL_glcm_MaximumProbability | 0.040105253 | Point mutations or duplications |
| wavelet.HHL_glcm_SumEntropy | 0.010788192 | Point mutations or duplications |
| wavelet.HHL_glrlm_RunVariance | 0.085859458 | Point mutations or duplications |
| wavelet.HHL_glrlm_ShortRunLowGrayLevelEmphasis | 0.068670852 | Point mutations or duplications |
| wavelet.HHH_glcm_JointAverage | 0.115708265 | Point mutations or duplications |
| wavelet.HHH_glcm_SumSquares | -0.095245237 | Point mutations or duplications |
| wavelet.HHH_gldm_SmallDependenceEmphasis | 0.03679868 | Point mutations or duplications |
| wavelet.LLL_glrlm_RunLengthNonUniformityNormalized | 0.048808146 | Point mutations or duplications |
| squareroot_firstorder_10Percentile | 0.0526717 | Point mutations or duplications |
| squareroot_glszm_LargeAreaHighGrayLevelEmphasis | 0.000599246 | Point mutations or duplications |
| squareroot_ngtdm_Strength | 0.000493645 | Point mutations or duplications |
| logarithm_glszm_LargeAreaHighGrayLevelEmphasis | 0.000145778 | Point mutations or duplications |
| logarithm_ngtdm_Strength | 0.000325827 | Point mutations or duplications |
| gradient_firstorder_10Percentile | -0.185797701 | Point mutations or duplications |
| lbp.2D_firstorder_Median | -0.06456406 | Point mutations or duplications |
| wavelet.HLL_gldm_SmallDependenceEmphasis | -0.038218377 | Point mutations or duplications |
| wavelet.HLH_glszm_LargeAreaHighGrayLevelEmphasis | -0.552503458 | Point mutations or duplications |
| lbp.2D_firstorder_InterquartileRange | 0.196468323 | Point mutations or duplications |
| wavelet.LHL_firstorder_Energy | 0.158038759 | Point mutations or duplications |
| wavelet.HHH_glcm_InverseVariance | -0.065994778 | Point mutations or duplications |
| b | -1.113977088 | deletions not involving codons 557/558 |
| original_shape_Elongation | 0.037160201 | deletions not involving codons 557/558 |
| original_shape_MeshVolume | 0.11473875 | deletions not involving codons 557/558 |
| original_shape_SurfaceVolumeRatio | 0.028281371 | deletions not involving codons 557/558 |
| wavelet.LLH_firstorder_TotalEnergy | 0.001131244 | deletions not involving codons 557/558 |
| wavelet.LLH_glcm_ClusterTendency | 0.080040223 | deletions not involving codons 557/558 |
| wavelet.LLH_glszm_GrayLevelNonUniformityNormalized | -0.095249417 | deletions not involving codons 557/558 |
| wavelet.LLH_glszm_GrayLevelVariance | 3.32E-07 | deletions not involving codons 557/558 |
| wavelet.LHL_glrlm_ShortRunHighGrayLevelEmphasis | -0.021215673 | deletions not involving codons 557/558 |
| wavelet.LHH_glrlm_HighGrayLevelRunEmphasis | -0.047288073 | deletions not involving codons 557/558 |
| wavelet.LHH_glrlm_LowGrayLevelRunEmphasis | 2.31E-05 | deletions not involving codons 557/558 |
| wavelet.LHH_glszm_ZoneEntropy | 0.009787937 | deletions not involving codons 557/558 |
| wavelet.HLL_glcm_Imc2 | -0.032641612 | deletions not involving codons 557/558 |
| wavelet.HLL_glcm_SumEntropy | 0.027450983 | deletions not involving codons 557/558 |
| wavelet.HLL_glrlm_ShortRunHighGrayLevelEmphasis | -0.246624919 | deletions not involving codons 557/558 |
| wavelet.HLH_firstorder_Kurtosis | -0.012952269 | deletions not involving codons 557/558 |
| wavelet.HLH_firstorder_Median | -0.03283257 | deletions not involving codons 557/558 |
| wavelet.HLH_firstorder_Minimum | -0.01867936 | deletions not involving codons 557/558 |
| wavelet.HLH_glcm_Contrast | -0.395660303 | deletions not involving codons 557/558 |
| wavelet.HLH_glcm_DifferenceAverage | -5.45E-15 | deletions not involving codons 557/558 |
| wavelet.HLH_glcm_SumEntropy | 0.064705248 | deletions not involving codons 557/558 |
| wavelet.HLH_glrlm_RunPercentage | -0.379897166 | deletions not involving codons 557/558 |
| wavelet.HLH_glrlm_ShortRunLowGrayLevelEmphasis | -0.253689488 | deletions not involving codons 557/558 |
| wavelet.HLH_gldm_HighGrayLevelEmphasis | -0.45815527 | deletions not involving codons 557/558 |
| wavelet.HLH_gldm_LargeDependenceEmphasis | 0.013443288 | deletions not involving codons 557/558 |
| wavelet.HLH_gldm_LowGrayLevelEmphasis | 0.005450698 | deletions not involving codons 557/558 |
| wavelet.HHL_glrlm_RunLengthNonUniformityNormalized | -0.043346141 | deletions not involving codons 557/558 |
| wavelet.HHH_firstorder_InterquartileRange | 0.011706019 | deletions not involving codons 557/558 |
| wavelet.HHH_glcm_Contrast | -0.314974876 | deletions not involving codons 557/558 |
| wavelet.HHH_glcm_Correlation | 0.36159495 | deletions not involving codons 557/558 |
| wavelet.HHH_glcm_DifferenceAverage | -0.049268474 | deletions not involving codons 557/558 |
| wavelet.HHH_glcm_Id | 0.00291224 | deletions not involving codons 557/558 |
| wavelet.HHH_glcm_Idn | 1.20E-06 | deletions not involving codons 557/558 |
| wavelet.HHH_gldm_HighGrayLevelEmphasis | -0.016817919 | deletions not involving codons 557/558 |
| wavelet.HHH_gldm_LowGrayLevelEmphasis | 1.30E-07 | deletions not involving codons 557/558 |
| square_firstorder_10Percentile | -0.067273649 | deletions not involving codons 557/558 |
| gradient_firstorder_Minimum | 0.059450891 | deletions not involving codons 557/558 |
| wavelet.LHH_glcm_DifferenceVariance | 0.351833872 | deletions not involving codons 557/558 |
| wavelet.HLL_gldm_SmallDependenceEmphasis | 0.153346554 | deletions not involving codons 557/558 |
| wavelet.LHL_firstorder_Energy | -0.212469901 | deletions not involving codons 557/558 |
| wavelet.HLH_gldm_LargeDependenceLowGrayLevelEmphasis | -1.920104769 | deletions not involving codons 557/558 |
| wavelet.HHH_glcm_InverseVariance | 2.015964845 | deletions not involving codons 557/558 |
| b | 0.096314344 | KIT exon 11 557/558 deletions |
| original_shape_Elongation | -0.083236689 | KIT exon 11 557/558 deletions |
| original_shape_SurfaceVolumeRatio | -0.08250722 | KIT exon 11 557/558 deletions |
| original_firstorder_Median | 0.028567345 | KIT exon 11 557/558 deletions |
| original_glszm_LargeAreaHighGrayLevelEmphasis | -0.062351771 | KIT exon 11 557/558 deletions |
| original_ngtdm_Strength | -0.00240708 | KIT exon 11 557/558 deletions |
| wavelet.LLH_glrlm_RunEntropy | 0.17918277 | KIT exon 11 557/558 deletions |
| wavelet.LLH_glrlm_ShortRunHighGrayLevelEmphasis | 0.132270246 | KIT exon 11 557/558 deletions |
| wavelet.LLH_glszm_GrayLevelNonUniformityNormalized | 0.130123405 | KIT exon 11 557/558 deletions |
| wavelet.LLH_glszm_GrayLevelVariance | -0.000158223 | KIT exon 11 557/558 deletions |
| wavelet.LLH_gldm_DependenceNonUniformityNormalized | 0.189188622 | KIT exon 11 557/558 deletions |
| wavelet.LLH_gldm_LargeDependenceLowGrayLevelEmphasis | 0.015606733 | KIT exon 11 557/558 deletions |
| wavelet.LLH_ngtdm_Coarseness | 0.015191307 | KIT exon 11 557/558 deletions |
| wavelet.LHL_glcm_Imc1 | 0.018653729 | KIT exon 11 557/558 deletions |
| wavelet.LHL_glszm_LargeAreaLowGrayLevelEmphasis | -0.824880526 | KIT exon 11 557/558 deletions |
| wavelet.LHL_gldm_SmallDependenceEmphasis | 0.040819988 | KIT exon 11 557/558 deletions |
| wavelet.LHL_gldm_SmallDependenceHighGrayLevelEmphasis | 0.015290378 | KIT exon 11 557/558 deletions |
| wavelet.LHH_firstorder_Mean | 0.045809264 | KIT exon 11 557/558 deletions |
| wavelet.LHH_firstorder_RootMeanSquared | 0.002940748 | KIT exon 11 557/558 deletions |
| wavelet.LHH_glcm_MCC | 0.008557617 | KIT exon 11 557/558 deletions |
| wavelet.LHH_glrlm_HighGrayLevelRunEmphasis | 0.13253663 | KIT exon 11 557/558 deletions |
| wavelet.LHH_gldm_SmallDependenceEmphasis | 0.11375448 | KIT exon 11 557/558 deletions |
| wavelet.HLL_firstorder_Mean | 0.072860782 | KIT exon 11 557/558 deletions |
| wavelet.HLL_firstorder_RootMeanSquared | 0.016632498 | KIT exon 11 557/558 deletions |
| wavelet.HLL_glszm_SmallAreaLowGrayLevelEmphasis | 0.073802375 | KIT exon 11 557/558 deletions |
| wavelet.HLH_firstorder_Kurtosis | -0.081087607 | KIT exon 11 557/558 deletions |
| wavelet.HLH_firstorder_Minimum | 0.072503803 | KIT exon 11 557/558 deletions |
| wavelet.HLH_firstorder_Skewness | 0.007536248 | KIT exon 11 557/558 deletions |
| wavelet.HLH_glcm_Imc1 | -0.054342819 | KIT exon 11 557/558 deletions |
| wavelet.HLH_glrlm_RunEntropy | 0.019207477 | KIT exon 11 557/558 deletions |
| wavelet.HHL_glcm_Imc2 | -0.006399923 | KIT exon 11 557/558 deletions |
| wavelet.HHL_glcm_MaximumProbability | -0.059702219 | KIT exon 11 557/558 deletions |
| wavelet.HHL_glrlm_LongRunHighGrayLevelEmphasis | -0.033076302 | KIT exon 11 557/558 deletions |
| wavelet.HHH_glcm_MaximumProbability | -0.134485846 | KIT exon 11 557/558 deletions |
| wavelet.HHH_glrlm_RunVariance | -0.201451107 | KIT exon 11 557/558 deletions |
| wavelet.HHH_glrlm_ShortRunHighGrayLevelEmphasis | 0.040887531 | KIT exon 11 557/558 deletions |
| wavelet.HHH_glszm_LargeAreaHighGrayLevelEmphasis | -0.038758553 | KIT exon 11 557/558 deletions |
| wavelet.HHH_glszm_ZoneVariance | -0.002668098 | KIT exon 11 557/558 deletions |
| wavelet.HHH_gldm_DependenceEntropy | -0.065050877 | KIT exon 11 557/558 deletions |
| square_firstorder_Median | 0.122302731 | KIT exon 11 557/558 deletions |
| square_glszm_LargeAreaEmphasis | -0.13533963 | KIT exon 11 557/558 deletions |
| square_glszm_LargeAreaHighGrayLevelEmphasis | -1.67E-16 | KIT exon 11 557/558 deletions |
| square_glszm_LargeAreaLowGrayLevelEmphasis | -1.43E-16 | KIT exon 11 557/558 deletions |
| squareroot_glszm_LargeAreaHighGrayLevelEmphasis | -0.056267245 | KIT exon 11 557/558 deletions |
| squareroot_ngtdm_Strength | -0.0022619 | KIT exon 11 557/558 deletions |
| logarithm_glszm_LargeAreaHighGrayLevelEmphasis | -0.001361886 | KIT exon 11 557/558 deletions |
| logarithm_ngtdm_Strength | -0.001924313 | KIT exon 11 557/558 deletions |
| exponential_firstorder_InterquartileRange | 0.032157329 | KIT exon 11 557/558 deletions |
| wavelet.LHH_glcm_DifferenceVariance | -0.207898158 | KIT exon 11 557/558 deletions |
| wavelet.HLL_gldm_SmallDependenceEmphasis | -0.44934538 | KIT exon 11 557/558 deletions |
| wavelet.HLH_glszm_LargeAreaHighGrayLevelEmphasis | 1.841358316 | KIT exon 11 557/558 deletions |

**Reference**

1. van Griethuysen JJM, Fedorov A, Parmar C, et al. Computational Radiomics System to Decode the Radiographic Phenotype. Cancer Res 2017;77:e104-e107.

2. Shrout PE, Fleiss JL. Intraclass correlations: uses in assessing rater reliability. Psychological bulletin 1979;86:420.

3. Friedman J, Hastie T, Tibshirani R. Regularization paths for generalized linear models via coordinate descent. Journal of statistical software 2010;33:1.
